# A Single-Event-Hardened Scheme for Ring Oscillator Applied to Radiation-Resistant PLL Microsystems

**DOI:** 10.3390/mi14040882

**Published:** 2023-04-19

**Authors:** Qi Xiang, Hongxia Liu, Yulun Zhou

**Affiliations:** Key Laboratory for Wide Band Gap Semiconductor Materials and Devices of Education Ministry, School of Microelectronics, Xidian University, Xi’an 710071, China; q77xiang@163.com (Q.X.); ylzhou_xd@163.com (Y.Z.)

**Keywords:** voltage-controlled oscillator, phase-locked loop, delay unit, single-event transient

## Abstract

A voltage-controlled oscillator (VCO) is one of the key modules of the phase-locked loop (PLL) microsystem, and it is easy to bombard using high-energy particles in a radiation environment, resulting in the single-event effect. In order to improve the anti-radiation ability of the PLL microsystems used in the aerospace environment, a new voltage-controlled oscillator hardened circuit is proposed in this work. The circuit consists of delay cells with an unbiased differential series voltage switch logic structure with a tail current transistor. By reducing sensitive nodes and using the positive feedback of the loop, the recovery process of the VCO circuit to the single-event transient (SET) is reduced and accelerated, so as to reduce the sensitivity of the circuit to the single-event effect. The simulation results based on the SMIC 130 nm complementary metal–oxide–semiconductor (CMOS) process show that the maximum phase shift difference of the PLL with the hardened VCO is reduced by 53.5%, which shows that the hardened VCO structure can reduce the sensitivity of the PLL to the SET and improve the reliability of the PLL in the radiation environment.

## 1. Introduction

With the continuous reduction in the feature size of integrated circuit devices and the rapid development of the aerospace industry, the phase-locked loop (PLL) operated in the radiation environment is seriously affected by the single-event effect, which cannot be ignored [1,2]. When high-energy particles act on the PLL, it will induce the single-event effect, which will make the frequency and phase of the output signal of the PLL drift and cause the circuit system function to be abnormal [3,4,5]. Each module of the PLL has a different response to the single-event effect [6,7]. Previous research results have proved that the charge pump (CP) and the voltage-controlled oscillator are sensitive modules of the PLL system, which are easily influenced by high-energy particles [8]. Therefore, using the radiation-hardened-by-design (RHBD) technology to reinforce the CP and voltage-controlled oscillator (VCO) can reduce the sensitivity of the PLL to the single-event effect [9,10]. The author has put forward an effective radiation hardening scheme for the CP in the previous work [11], and this work focuses on the radiation hardening design of the VCO module.

In order to improve the ability of the VCO to resist the single-event effect, many researchers have proposed effective VCO-hardened structures [12,13,14,15,16,17,18,19]. H. Yuan et al. reduced the sensitivity of the VCO to bias through multi-bias technology and interleaving bias [12], while the ability of the single-event transient effect tolerance of the ring oscillator was improved through layout hardening. The drawbacks of this scheme were a significant increase in area and high power consumption. P. Rajalingam et al. presented a radiation-hardened current-starved inverter buffer for the VCO [13]. The proposed VCO was composed of the current-starved inverter delay VCO and majority voter. R. Kumar et al. put forward a scheme of oscillator redundancy reinforcement [14], which used two independent ring oscillators to ensure that when one of them was affected by radiation, the system could control the other to compensate. The redundant reinforcement structure effectively improved the anti-radiation ability of the PLL, but the chip area and power consumption were also significantly increased. Z. Chen and others improved the delay cell of the VCO [15], and used a cross-coupled symmetrical load delay cell to form the circuit of the hardened oscillator. The positive feedback of the improved delay cell was used to speed up the recovery process of the circuit affected by the single-event transient (SET). The circuit structure of the hardened scheme was simple, but the sensitive nodes in the bias circuit still exist. S. M. Jung et al. used an additional feedback voltage-controlled oscillator to filter the voltage fluctuation on the control voltage [16], which effectively reduced the response of the single-event transient effect in the PLL, but the PLL circuit was complex and difficult to realize. H. Liu et al. improved the bias circuit by digital coding [17], and adopted the idea of digital processing to filter out the influence of the transient pulse and keep the bias voltage stable. However, this digital coding reinforcement scheme would introduce additional sensitive nodes in digital circuits. B. Liang et al. proposed a proportional and integral path-independent PLL-hardened scheme [18], and simulation results showed that the frequency error caused by radiation on the VCO was reduced by 80% to 90%. V. Diez-Acereda and others used a filter network to harden the oscillator [19], and introduced an RC network to suppress current transients, thus improving the ability of the VCO to resist the SET effect. However, using large resistors and capacitors could increase the layout area. At present, the reinforcement design of a voltage-controlled oscillator mainly starts with the following improvement ideas: improving the structure of the delay cell and bias circuit itself, adopting a multi-mode hardened circuit, and node filtering. Among them, the multi-mode hardened structure has the best reinforcement effect, but its power consumption and layout area are doubled.

In this work, a low-cost VCO reinforcement structure applied to the PLL is proposed, and system-level simulation and circuit-level single-event simulation are carried out under the standard 130 mm CMOS process, which verifies the ability of this structure to suppress the SET. Compared with related work, the VCO proposed in this work has the advantages of fewer SET-sensitive nodes, a small area, low power consumption, and easy circuit implementation. The next section describes the topology of the PLL and analyzes the influence of the SET on the VCO. The third part describes the circuit of the delay unit of the hardened VCO in detail. The fourth part is the simulation of the SET effect of the PLL before and after hardening and the analysis of the simulation results. The last part is the summary of the whole work.

## 2. Responses to the SET in the VCO

### 2.1. Topology of the CPPLL

**Figure 1:**
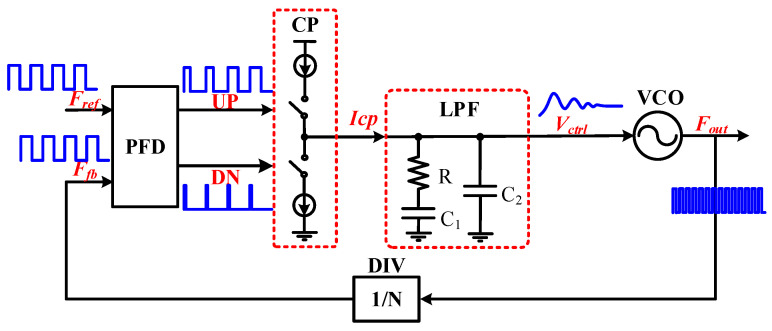
The topology of the CPPLL.

The phase-locked loop is a feedback control system, Figure 1 shows the topological structure of the charge pump phase-locked loop (CPPLL), which is mainly composed of the five submodules, namely, the frequency discriminator (PFD), the CP, the low-pass filter (LPF), the VCO, and the frequency divider (DIV).

The PFD module compares the phase difference and frequency difference of the periodic input reference clock signal *F_ref_* and the frequency divider feedback signal *F_fb_* into the corresponding pulse signals UP and DN. The CP module selects the working mode according to the pulse signal, charges and discharges the capacitor in the loop filter, converts it to DC level after filtering, and controls the VCO frequency, thereby reducing the phase difference between *F_ref_* and *F_fb_*. With the infinite system gain of the PFD/CP, the oscillator control voltage can only remain stable when the phase difference between *F_ref_* and *F_fb_* is zero. Otherwise, the LPF capacitor will adjust the output frequency of the VCO through continuous charging and discharging. Finally, when the PLL is locked, the phases of *F_ref_* and *F_fb_* are consistent, and the PLL outputs a continuous and stable *N* × *F_fb_* frequency signal.

### 2.2. Analysis of the SET’s Impact on the VCO

The VCO is the core module of the PLL, and it is also sensitive to the SET effect [20]. This section will simulate the response of the single-event effect in the VCO through different bombardment nodes, different bombardment times, and different bombardment energies, and study its action trend and failure mechanism.

#### 2.2.1. SET Current Pulse Model

In the circuit-level simulation of the single-event effect, a transient current pulse is connected in parallel to a sensitive node in the circuit to simulate the response of the SET effect. In this work, the double-exponential current pulse model is used to simulate the radiation response of the high-energy particles to the PLL. This model simulates the pulse current shape formed by the single-event effect at the sensitive position of the semiconductor device in the form of the double-exponential function [21], which is the most widely used pulse current source model in the circuit-level single-event effect. The expression formula of the double-exponential current is:

(1) $$\left\{ \begin{array}{l} I(t)=I_{o}\left(e^{-\frac{t}{\alpha }}-e^{-\frac{t}{\beta }}\right)\\ I_{o}=\frac{Q_{\mathrm{tot}}}{\alpha -\beta } \end{array}\right.$$

In Formula (1), $Q_{tot}$ is the total charge ionized when particles bombard the transistor, $I_{o}$ is the peak value of the transient current pulse, α is the time constant of charge collection, and β is the initial time constant of charge generation. To ensure the accuracy of the single-event effect (SEE) circuit-level simulation, SEE device-level simulation data are used for fitting and determining model parameters. The device structure model under SMIC 130 nm process conditions was constructed by using the device-level simulation software Sentaurus TCAD 2020, and the model was simulated by the single-event effect, and the obtained SET transient pulse current data were fitted to determine the unknown value of the double-exponential model function. In this work, a 500 fC deposition charge was used to verify the anti-SET performance of the strengthened structure. The peak $I_{o}$ of the transient pulse current of the double-exponential model was 2.14 mA, the charge collection constant α was 216 ps, and the initial time constant β was 12 ps.

#### 2.2.2. SET Response of Different Bombardment Nodes

**Figure 2:**
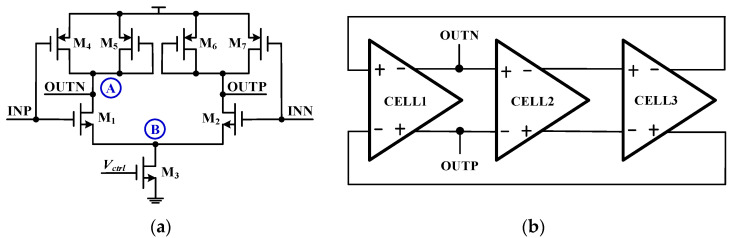
SET circuit-level simulation for different nodes and delay cells of the VCO. (**a**) Different nodes. (**b**) Different delay cells.

Due to the different delay cell structures, each node in the VCO will respond differently to the single-event effect. According to the circuit structure of the voltage-controlled oscillator, the nodes A and B of the three delay cells of the ring oscillator circuit were bombarded with a 500 fC deposition charge. As shown in Figure 2, different sensitive nodes and different delay units of the traditional VCO were simulated by high-energy particle bombardment. Among them, the INP and INN were the differential inputs of the delay unit, and the OUTP and OUTN were the differential outputs of the delay unit.

**Figure 3:**
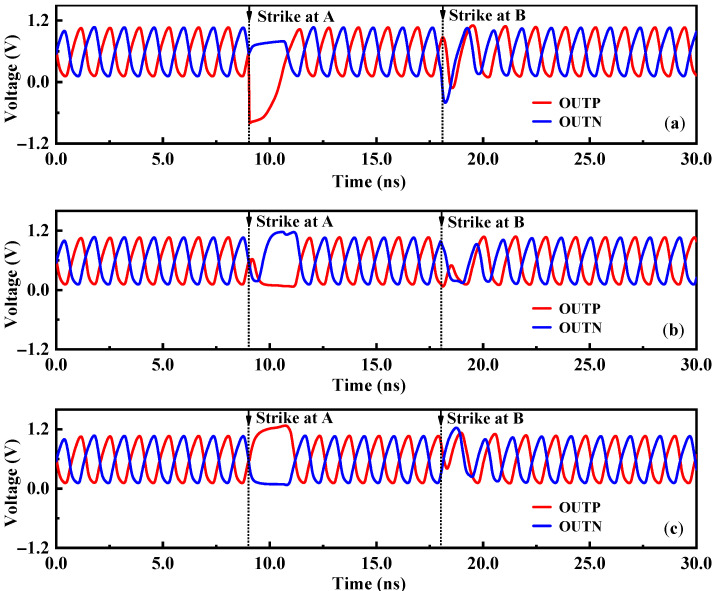
Simulation results of the SET responses of different nodes and different delay units in the VCO: (**a**) bombardment of CELL1; (**b**) bombardment of CELL2; and (**c**) bombardment of CELL3.

**Figure 4:**
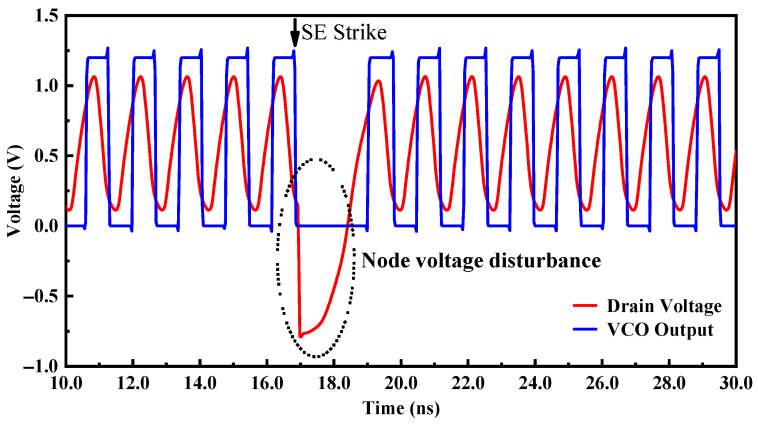
Simulation results of the node voltage disturbance and the VCO output.

Figure 3 shows the simulation results of different nodes and different delay cells of the VCO after being bombarded by a SET. Observing the simulation results of the differential output terminals OUTP and OUTN of the delay unit, it was found that the node A in each delay cell was the most sensitive to the SET effect, while node B was less sensitive to the SET, so the sensitive node was the drain of the output transistor. The main reason was that transistors M_1_ and M_2_ contributed almost half of the bias current, so the extra SET current may introduce more phase shift besides amplitude fluctuation, so node A was sensitive to the single-event effect. Node B was located at the node of the drain of the tail current transistor and the differential common mode equivalent ground. When the loop works at high frequency, the tail current is large, and the differential circuit has good common-mode noise suppression ability, which makes node B less sensitive to the single-event effect. At the same time, the working voltage of node B itself was small, and the voltage drop amplitude was small when it was bombarded by particles, so the voltage disturbance of node B has little influence on the output waveform. Bombarding CELL1 mainly affects the amplitude and phase of the output signal, while bombarding CELL2 and CELL3 mainly affects the phase of the output signal. The amplitude disturbance at CELL2 and CELL3 nodes was weakened after passing through the delay loop, showing a small amplitude disturbance. The reason why the oscillation waveform changes after being bombarded by high-energy particles was that, as shown in Figure 4, when the SET current acts on the sensitive node, the charge was deposited and stored on the node, and the voltage on the node would remain disturbed, eventually leading to the failure of oscillation. The voltage disturbance was eliminated until the charge at the node dissipated.

#### 2.2.3. SET Response of Different Bombardment Times

**Figure 5:**
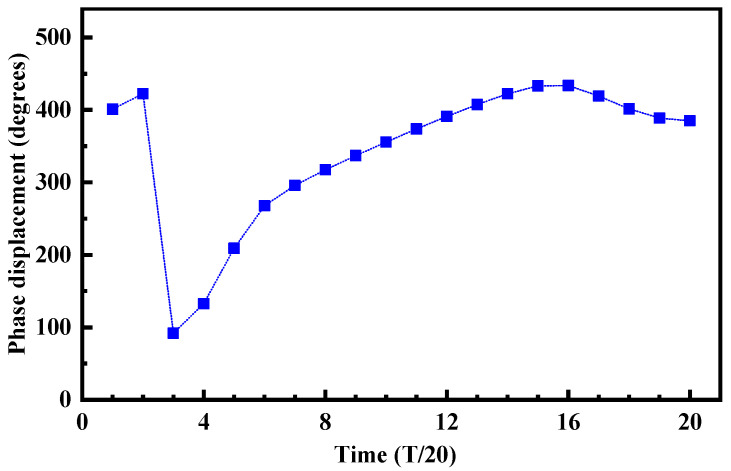
Simulation results of maximum phase difference of output clocks of different bombardment times.

Because the VCO of the PLL was in a continuous oscillation state and the output oscillation waveform was periodic, the response of the differential ring oscillator to the single-event current pulse was also periodic. The SET effect was different under different particle bombardment times, so it was necessary to study the influence of different bombardment times on the SET response in the VCO. The oscillation frequency of the VCO was 720 MHz, and the bombardment node selected output node A of CELL1. In a complete oscillator output signal period, 20 time points were evenly selected as the bombardment time points of particles, simulating the response of the drain of transistor M_1_ after being bombarded by high-energy particles at different bombardment times, and the maximum phase difference change was plotted on the curve shown in Figure 5.

**Figure 6:**
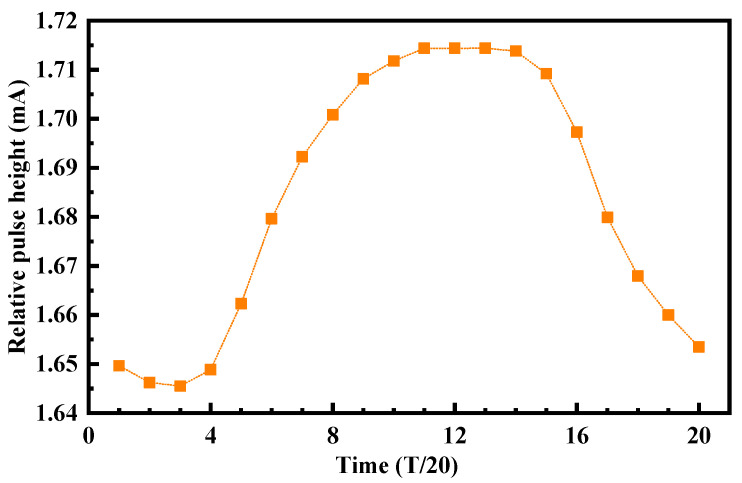
Simulation results of current pulse height of different bombardment times.

The simulation results in Figure 5 show that the SET effect response of the VCO has time-varying characteristics. When the delay cell of the VCO was bombarded at different times, the maximum phase difference of the VCO output signal was different, the maximum phase difference was 433.53°, and the minimum phase difference was 92.025°. It shows that the SET effect has different effects on the oscillation of the VCO when the transistor was in different working states. The main reasons why the SET effect of the VCO has a time-varying response were that the phase difference value was directly related to the voltage gain at the bombardment time, and it was also affected by the amplitude and pulse width of the SET current, the internal structure of the circuit, the signal change trend, and the transient direction of the SET current, so the response of the sensitive node to different particle bombardment times was different. Figure 6 also shows that bombarding the delay cell at different times would produce the SET current pulses with different heights. The results show that with the increases in transistor drain voltage, the height of the SET pulse also increases gradually. The main reason was that when the node was bombarded by high-energy particles, the charge accumulation at the node mainly depends on the drift motion of the funnel region, and the speed of charge accumulation at the node after bombardment depends on the electric field of the drain depletion layer, which in turn depends on the size of the drain voltage. The larger the oscillation waveform of the VCO, the greater the drain voltage, and the faster the charge accumulation of the bombarded node, the higher the peak value of the SET pulse current of the node.

#### 2.2.4. Response to the SET with Different Bombardment Energies

**Figure 7:**
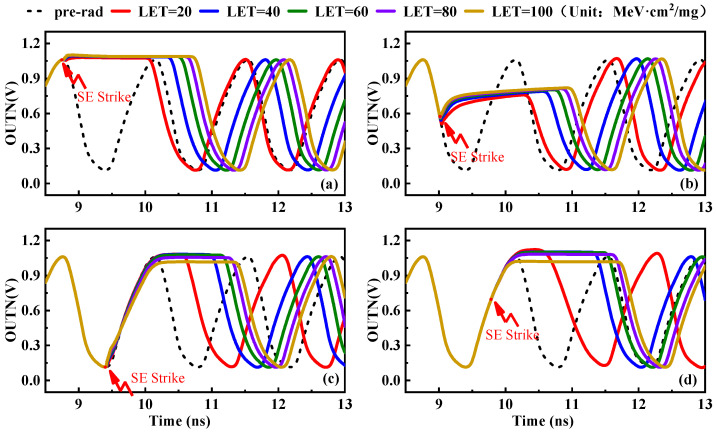
Simulation results of the response to the SET with different bombardment energies in the VCO. (**a**) Bombardment at the peak. (**b**) Bombardment when the wave falls. (**c**) Bombardment at the trough. (**d**) Bombardment when the wave rises.

When particles with different energies bombard a semiconductor device, the number of ionized electron–hole pairs in the semiconductor device was different, and the amount of charge accumulated at the drain was also different, so the SET response on the transistor was also different. Four special times of the oscillation period were selected to bombard the positive output node of CELL1, and the SET response of the negative output node of the VCO under different bombardment energy was analyzed. The simulation results of the response to the SET with different bombardment energies and different bombardment times in the VCO are shown in Figure 7.

The simulation results in Figure 7 show that the maximum phase difference of the VCO response was increased with the increase in bombardment energies. In the four bombardment times of the oscillator, the SET response of the VCO was increased with the increase in bombardment energies, and the influence on the oscillation waveform also increases. The results show that the SET response of the VCO was more obvious with the increase in bombardment energies. The main reason was that with the increase in particle bombardment energies, the amount of charge deposited on the bombarded node also increases, which leads to the single-event effect having a greater impact on the output signal frequency of the voltage-controlled oscillator. The reinforcement structure can consider reducing the charge accumulation of the nodes and accelerating the single-event response of the nodes.

## 3. The Proposed SET-Hardened VCO Circuit

**Figure 8:**
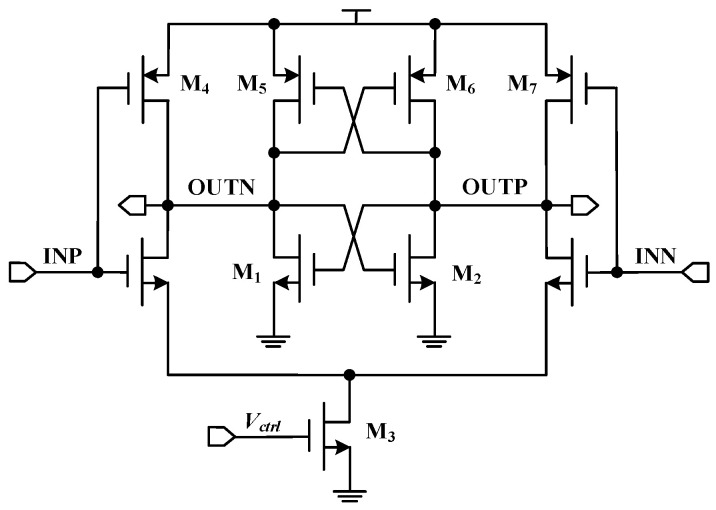
The proposed SET-hardened VCO.

The VCO is the core module of the CPPLL. As the analog circuit and output circuit of the CPPLL, the VCO is sensitive to the SET effect, and its reinforcement performance will directly affect the frequency locking of the output signal of the CPPLL. According to research in the literature [22], the differential cascaded voltage switching logic (DCVSL) has a good ability to resist the single-event effect, so when designing the VCO reinforcement structure, the delay cells of the VCO combine the latch structure and DCVSL structure. The circuit did not add additional circuit modules, only a few transistors based on the original delay cell structure, and obtains better radiation resistance without sacrificing area and frequency characteristics. The circuit structure of the single-event-hardened delay cell proposed in this work is shown in Figure 8. A delay cell was composed of two inverters with differential cascade voltage switching logic. The control voltage adjusts the equivalent resistances of the output nodes OUTN and OUNP by controlling the working current of the current transistor M_3_, and controls the delay time of each stage of the delay circuit, thus modulating the VCO output oscillation frequency. Compared with the original delay cell structure, two NMOS transistors were added, and the original diode-connected PMOS transistors were changed to gate-drain cross-connection. Two back-to-back inverter structures were formed, and a positive feedback loop was formed. When high-energy particles bombard the output node, the recovery process of the circuit to the SET effect can be accelerated under the action of the positive feedback, which makes the circuit have a certain anti-SET ability and did not introduce additional bias nodes that were sensitive to the SET. Although the tail current transistor drain node still exists, the SET effect generated at this node would be consumed by the delay unit, showing insensitivity to the SET.

## 4. Simulation and Result Analysis

**Figure 9:**
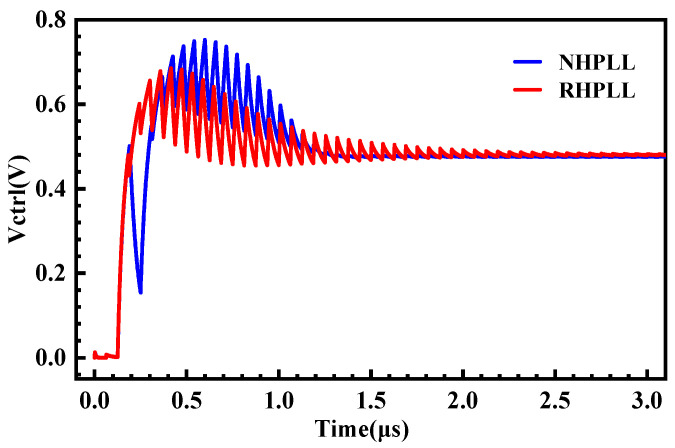
Locking processes of the RHPLL and NHPLL.

In order to verify the radiation resistance of the proposed VCO-hardened structure, an unhardened PLL (NHPLL) and a hardened PLL (RHPLL) were built by the SMIC 130 nm CMOS process. The two PLL systems were different except for the delay cell of the VCO, and the other circuit modules were identical. Through the overall simulation of the PLL before and after hardening, the locked state as shown in Figure 9 was obtained. The simulation results showed that the locking times of the NHPLL and RHPLL were 1.5 μs and 2.7 μs, respectively, and the loop performance of the VCO-hardened PLL system had hardly changed.

**Figure 10:**
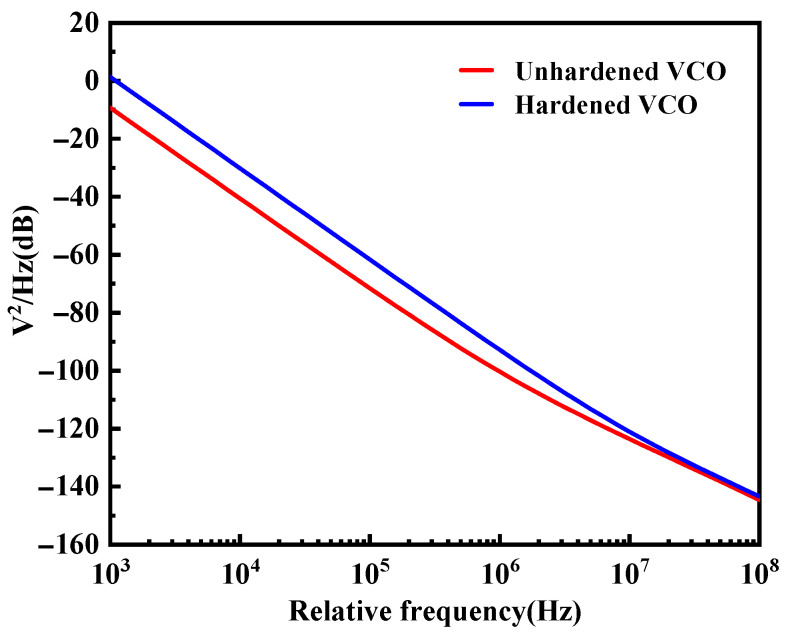
Simulation results of the phase noise of the VCO before and after hardening.

The VCO phase noise was the main source of the out-of-band noise in the PLL system, and it was also a key parameter to measure the VCO performance. The phase noise performance of the VCO before and after hardening was simulated, and a comparison of the phase noise effect is shown in Figure 10. The results show that the hardened VCO circuit had better phase noise performance.

**Figure 11:**
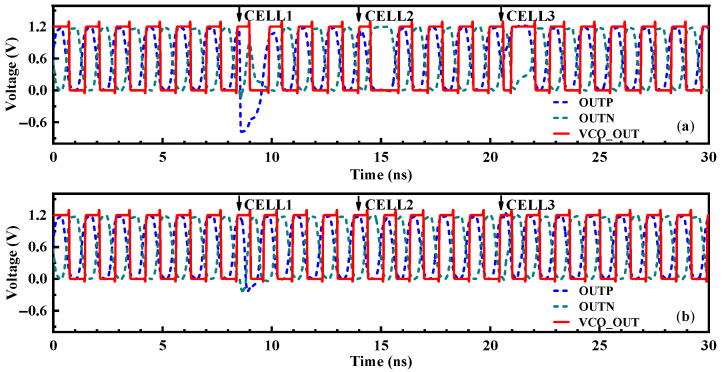
Simulation results of node voltage disturbance of the VCO delay cell. (**a**) Strike at node A. (**b**) Strike at node B.

**Figure 12:**
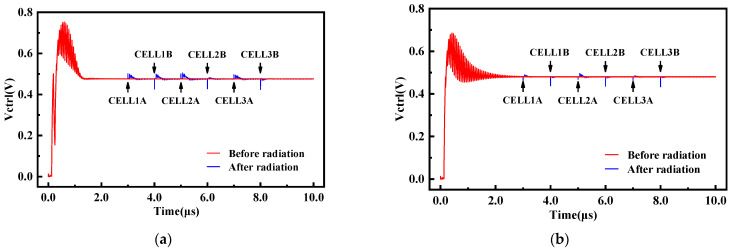
Simulation results of the PLL control voltage disturbance. (**a**) Unhardened VCO. (**b**) Hardened VCO.

When the phase-locked loop was in the fully locked state, the impact of high-energy particles on nodes A and B in each delay cell of the VCO were simulated by adding a SET current transistor. The simulation results of the VCO delay cell node voltage disturbance and the PLL control voltage disturbance are shown in Figure 11 and Figure 12. The results showed that the sensitivity of the reinforced VCO to the SET was reduced. Node B was almost insensitive to the SET effect, while node A was a little sensitive to the SET effect. The reinforced VCO had better radiation resistance to node B of each delay cell and better radiation resistance to delay cells CELL2 and CELL3. The maximum phase difference of the VCO output signal in the worst case was 193.36°, which was 55.4% lower than that before reinforcement, which showed that the reinforced structure had certain radiation resistance. However, the transient voltage pulse formed by the SET effect cannot be greatly weakened only by the delay cell of the last stage. Compared with the NHPLL, the RHPLL adopts the circuit structure of the hardened VCO, which realizes a smaller maximum phase difference, smaller control voltage disturbance, and a shorter PLL recovery time, effectively reducing the sensitivity of the VCO delay cell nodes to the single-event effect and improving the anti-radiation ability of the CPPLL.

**Figure 13:**
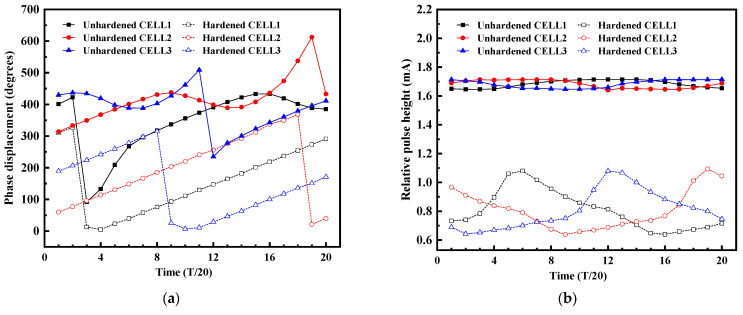
Performance comparison results of the three delay cells at different times in the same cycle before and after hardening. (**a**) Comparison result of the phase error. (**b**) Comparison result of the SET current pulses.

In order to more clearly demonstrate the hardened performance of the proposed VCO, the phase error and pulse current of three delay cells at different times in the same oscillation cycle before and after hardening were simulated, and the error effects of the three delay cells before and after hardening were compared as shown in Figure 13. Figure 13a showed the comparison results of the phase errors before and after hardening. After hardening, the phase errors of the three delay cells were significantly reduced, and the proposed hardened structure effectively relieves the response of the single-event effect to the VCO phase. The average value of the phase error improvement obtained by the simulation of the delay unit at different times in a cycle was solved, and the average values of the phase error improvement of the three delay units were 57.8%, 53.5%, and 59.3%. Therefore, the improvement degree of 53.5% in the worst case was taken as the improvement effect of the proposed hardened structure. Bombarding the delay cell at different times would produce the SET current pulses with different heights. Figure 13b showed the comparison results of the SET pulse current before and after hardening. The SET current before hardening was maintained at 1.7 mA, while the SET current after hardening was reduced to less than 1 mA. The sharp decrease in the SET pulse current made the influence of the radiation effect on the VCO obviously reduced, which showed that the hardened structure had high radiation resistance.

**Figure 14:**
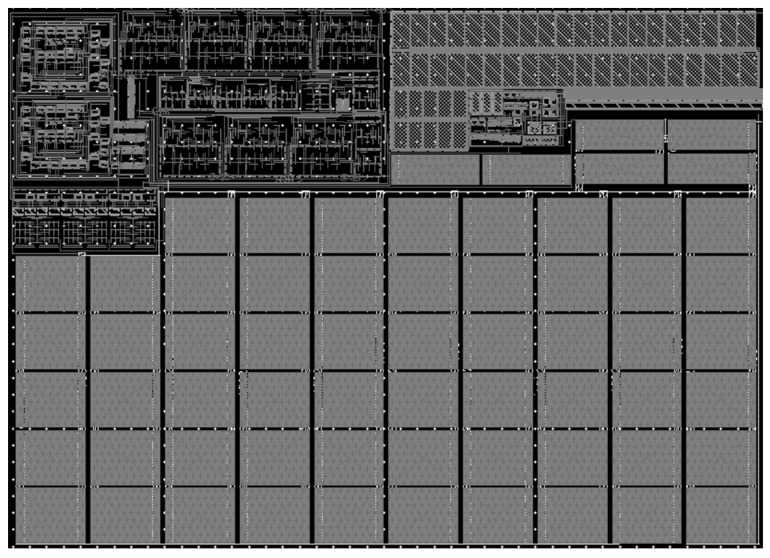
Layout of the hardened PLL.

The function and performance of analog circuits will be affected by the layout. In this work, a 0.13 μm CMOS process was used to complete the layout design of each module and the whole reinforced PLL, as shown in Figure 14. The overall layout of the PLL was reasonable and compact, and the layout was rectangular with an area of 158 μm × 222 μm. Compared with the VCO before reinforcement, the layout area of the reinforced VCO was almost unchanged.

The loop performance of the CPPLL with the hardened VCO was simulated comprehensively, and the system index parameters obtained by the simulation test were recorded in Table 1. The simulation data showed that, compared with the NHPLL, the output frequency and charge pump current of the RHPLL were unchanged; the gain of the VCO, the ripple of control voltage *V_ctrl_*, and the locking time of the CPPLL were slightly increased; the phase noise of the VCO was slightly reduced; and the area and power consumption of the VCO were slightly increased. It showed that the hardened VCO had a certain anti-single-event effect without sacrificing a large area and power consumption. The main reasons for the changes in the VCO gain, the VCO phase noise, and the locking time were the changes in the VCO delay cell structure. To sum up, the RHPLL used a relatively simple circuit to improve the anti-radiation performance of the VCO without sacrificing a large system index, and improved the reliability of the PLL in the irradiation environment.

Table 2 lists the comprehensive comparison between the hardened VCO structure proposed in this study and the advanced VCO against the SET effect in other studies. Other literature had achieved a certain hardened effect at the expense of a larger area. The delay cell structure designed for voltage-controlled oscillator reinforcement in this work had a good ability to resist the single-event effect, and the control voltage directly acted on the delay cell without using bias nodes and other SET-effect-sensitive nodes. The circuit structure was simple and easy to implement, and the structural area and power consumption of the hardened oscillator were almost unchanged.

## 5. Conclusions

In order to improve the reliability of the PLL in the aerospace environment, a single-event-effect-reinforced VCO circuit structure was proposed. The positive feedback of the loop was used to speed up the recovery process of the SET effect and reduce the sensitivity of the VCO to the SET. Based on SMIC 130 nm CMOS simulation, the maximum phase difference was reduced by 53.5%. Compared with the traditional phase-locked loop anti-radiation reinforcement circuit, a reinforcement structure with a smaller area, lower power consumption, and simpler circuit implementation was used to achieve more effective anti-radiation performance.

## Figures and Tables

**Table 1:** Performance comparison of the PLL before and after hardening.

Performance Parameter		NHPLL	RHPLL
Supply voltage		1.2 V	1.2 V
Output frequency		0.72 GHz	0.72 GHz
CP working current		20 μA	20 μA
VCO gain		668.86 MHz/V	687.16 MHz/V
Phase noise @1 MHz		99 dBc/Hz	92 dBc/Hz
Control voltage ripple		0.20 mV	0.64 mV
Lock time @0.72 GHz		1.5 μs	2.7 μs
Power consumption of the VCO		716.8 μW	799.6 μW
Area of the VCO		17.8 μm × 42 μm (1×)	19.4 μm × 42 μm (1.08×)
SET response	*V_ctrl_* fluctuation peak	25 mV	6.8 mV
	Recovery time	1154.4 ns	314.8 ns
	Maximum phase error	433.53°	193.36°

**Table 2:** Comparison with other works.

Parameter	Reference [19]	Reference [23]	This Work
Technology node	180 nm CMOS	65 nm CMOS	130 nm CMOS
Frequency	2.4 GHz	4.8 GHz	720 MHz
Deposited charge or LET	600 fC (≈60 MeVcm^2^/mg)	570 fC (≈57 MeVcm^2^/mg)	500 fC (≈50 MeVcm^2^/mg)
Circuit area	Increased by 1 time	Increased by 3 times	Increased by 9.0%
Power consumption	Increased by 14.6%	Declined by 28.3%	Increased by 11.5%
Circuit	Ring oscillator circuit	LC oscillator circuit	Ring oscillator circuit
Maximum phase error	Declined by 50%	Declined by 74.2%	Declined by 53.5%

## Data Availability

Not applicable.
